# Exploring Middle School Students’ Perspectives on Using Serious Games for Cancer Prevention Education: Focus Group Study

**DOI:** 10.2196/31172

**Published:** 2022-01-24

**Authors:** Olufunmilola Abraham, Lisa Szela, Mahnoor Khan, Amrita Geddam

**Affiliations:** 1 Social & Administrative Sciences Division School of Pharmacy University of Wisconsin-Madison Madison, WI United States; 2 School of Pharmacy University of Wisconsin-Madison Madison, WI United States

**Keywords:** adolescents, adolescent education, adolescent health, older children, middle school students, cancer awareness, cancer education, cancer prevention, health education, serious games

## Abstract

**Background:**

Cancer in the United States is a leading cause of mortality. Educating adolescents about cancer risks can improve awareness and introduce healthy lifestyle habits. Public health efforts have made significant progress in easing the burden of cancer through the promotion of early screening and healthy lifestyle advocacy. However, there are limited interventions that educate the adolescent population about cancer prevention. Previous studies have demonstrated the effectiveness of serious games (SGs) to teach adolescents about healthy lifestyle choices, but few research efforts have examined the utility of using SGs to educate youth specifically on cancer prevention.

**Objective:**

This study aimed to investigate middle school students’ preferences for the use of SGs for cancer prevention education. The study also characterized the students’ perceptions of desired game design features for a cancer prevention SG.

**Methods:**

Focus groups were held to allow adolescents to review a game playbook and discuss gaming behaviors and preferences for an SG for cancer education. The game playbook was developed based on “Cancer, Clear & Simple,” a curriculum intended to educate individuals about cancer, prevention, self-care, screening, and detection. In the game, the player learns that they have cancer and is given the opportunity to go back in time to reduce their cancer risk. A focus group discussion guide was developed and consisted of questions about aspects of the playbook and the participants’ gaming experience. The participants were eligible if they were 12 to 14 years old, could speak and understand English, and had parents who could read English or Spanish. Each focus group consisted of 5 to 10 persons. The focus groups were audio recorded and professionally transcribed; they were then analyzed content-wise and thematically by 2 study team members. Intercoder reliability (kappa coefficient) among the coders was reported as 0.97. The prevalent codes were identified and categorized into themes and subthemes.

**Results:**

A total of 18 focus groups were held with 139 participants from a Wisconsin middle school. Most participants had at least “some” gaming experience. Three major themes were identified, which were educational video games, game content, and purpose of game. The participants preferred customizable characters and realistic story lines that allowed players to make choices that affect the characters’ outcomes. Middle school students also preferred SGs over other educational methods such as lectures, books, videos, and websites. The participants desired SGs to be available across multiple platforms and suggested the use of SGs for cancer education in their school.

**Conclusions:**

Older children and adolescents consider SGs to be an entertaining tool to learn about cancer prevention and risk factors. Their design preferences should be considered to create a cancer education SG that is acceptable and engaging for youth.

## Introduction

Cancer is a serious disease that affects the health, well-being, and overall quality of life for the diagnosed person as well as their loved ones. Despite progress in medical and scientific technology, cancer remains the second leading cause of mortality in the United States [1]. The American Cancer Society estimates that 1.9 million new cancer cases will be diagnosed in the United States in 2021 [1]. In addition to the personal health and emotional costs of cancer, it is also a leading American health care expenditure, projected to cost $245 billion by the year 2030 [2]. While the type of cancer, stage, and diagnosed individual’s age can affect prognosis and outcomes, cancer is a severe illness that significantly affects people’s lives across the United States.

Although the adolescent population is not the most at risk for cancer mortality, adolescence is an important stage for cancer prevention [3]. Adolescents are in a crucial developmental phase where they can be influenced to develop healthy habits, such as eating a healthy diet and exercising or avoiding hazardous habits, such as smoking and vaping [4]. Behaviors developed in adolescence can reduce cancer risk or predispose adolescents to cancers at later stages in life. In 2014, approximately 42% of diagnosed cancers (excluding nonmelanoma skin cancers) and 45.1% of cancer deaths were attributed to modifiable risk factors including cigarette smoking, excess body weight, alcohol intake, poor diet, physical inactivity, ultraviolet light exposure, and cancer-associated infections [5]. Targeting youth for early cancer education related to modifiable cancer risk factors can promote healthy lifestyle patterns that remain throughout the rest of their adult lives.

In recent years, researchers have explored serious games (SGs) as an educational public health tool due to the popularity of gaming among teenagers and young adults. The Pew Research Center reports that 80% of teenagers have access to gaming devices, and 90% play video games [6]. SGs, also known as educational games, are video games designed not only for entertainment, but to educate or create awareness of a certain issue [7]. Between 2003 and 2014, 16 SGs were developed to promote vaccinations and demonstrated the potential to influence health behaviors [8]. SGs have also been used to educate students about healthy eating habits. In 2010, a meta-analysis analyzed the role of 11 video games designed to support children with type 1 diabetes mellitus in managing their disease state [9]. These games presented education in a comfortable, exciting, and understandable manner and demonstrated the potential to educate students. Additionally, SGs have been used to educate adolescents on medication use [10]. These studies suggest that SGs can improve health literacy in adolescents and indicate the potential for positive impact on improving adolescent awareness regarding cancer prevention.

While studies regarding SG cancer medication education in diagnosed adolescents have been conducted in the United States, there are few studies that examine the role of SGs for cancer prevention strategies and associated cancer risk factors for adolescents [11]. In a recent study, researchers conducted a randomized controlled trial to study the educational impact of a web-based game intervention (Re-Mission [Hopelab]) on cancer risk perception in college students and the relationship between risky behaviors and carcinogenic susceptibility. The results indicated that SGs can have an impact on information-seeking behaviors and perceptions of cancer among young adults [12]. This strategy could be implemented in SGs targeted toward adolescents to reduce their risk of cancer.

Public health efforts have significantly improved cancer awareness, preventative screening, and lifestyle modification among adults [13,14]. However, there are few SGs specifically developed to educate the adolescent population about the importance of cancer prevention [15]. Exploration of this emerging field can provide insights on the impact of SGs and game features preferred by older children and adolescents. Investigating the use of SGs in cancer education is crucial, as education can instill healthy habits to prevent future cancer risk behaviors. Thus, this study aimed to investigate middle school students’ preferences for the use of SGs to provide cancer prevention education and their desired game design features for a cancer prevention SG.

## Methods

### Study Design

Focus groups were chosen to capture group interactions, discussions of participants’ gaming behaviors, and preferences for a cancer education game [16]. This qualitative data collection method allowed the participants to expand on their responses and opinions and offered the moderators an opportunity to ask follow-up questions as needed. A focus group discussion guide was created by the study team based on a questionnaire from the principal investigator’s previous research (Multimedia Appendix 1). The study team reviewed and revised the discussion guide prior to data collection. The guide consisted of mostly open-ended questions about aspects of a cancer education SG playbook. Questions were designed to explore the participants’ perspectives on characters, story line and scenarios, and the purpose of the game, as well as their experience with video games. The participants’ demographic information was collected, including age, gender, race and ethnicity, zip code, and number of persons under 18 years living in their household.

### Game Playbook

The game playbook used in the focus group discussions was created based on “Cancer, Clear & Simple,” a curriculum designed by the Cancer Health Disparities Initiative to educate individuals about cancer [17,18]. The curriculum covers “Cancer Basics,” “Cancer Prevention & Self-Care,” and “Cancer Screening & Detection” and has been adapted for rural, Black, and Latino communities [17,18].

The game playbook was presented to older children and adolescents in paper format and included images, a brief game overview, and descriptions of game levels. The participants reviewed the game playbook before answering focus group questions about the overall game and individual aspects of the game. The playbook introduced a scenario in which the player is a 57-year-old patient who learns of a stage 4 colorectal cancer diagnosis. The player is then transported back in time and given the opportunity to make different life choices. If the player makes healthier choices regarding diet, tobacco use, and cancer screening, they are transported back to the present day, where they find they now have stage 2 colorectal cancer and an increased chance of survival.

**Figure 1:**
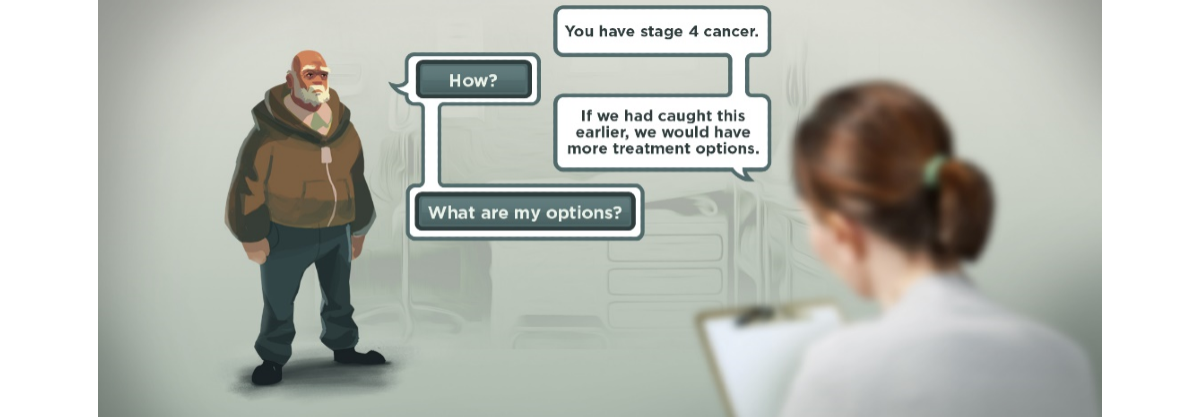
Level 1 of the game playbook.

The game playbook presented to the participants included 4 levels with corresponding images and level descriptions. In Level 1, the player is introduced to the game, and their doctor informs them that they have stage 4 colorectal cancer (Figure 1).

**Figure 2:**
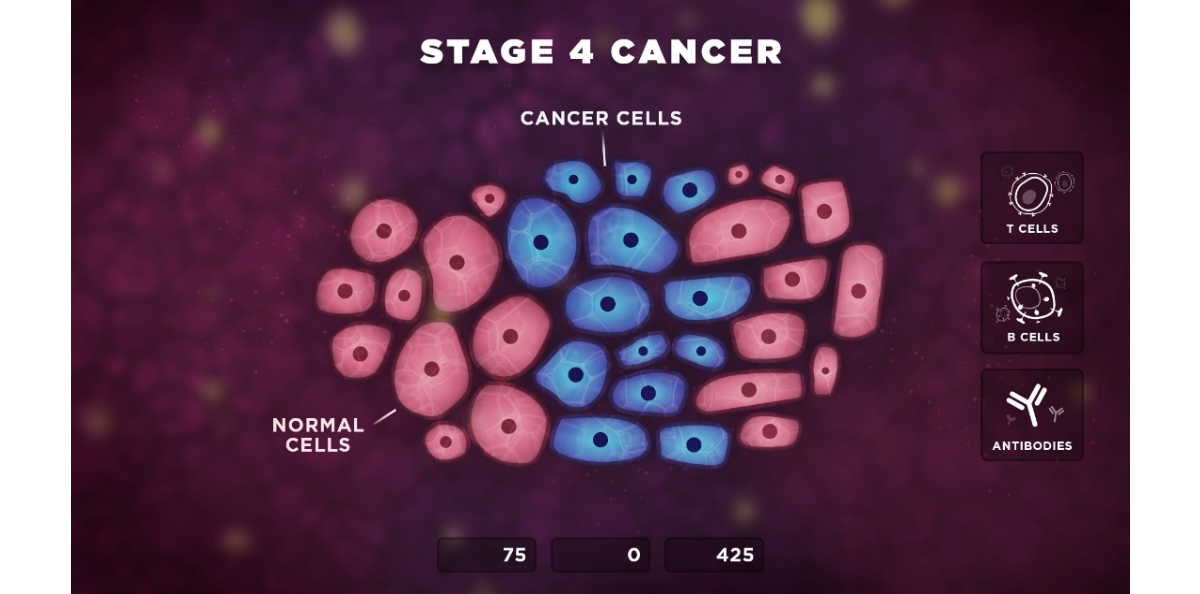
Level 2 of the game playbook.

The purpose of Level 1 was to explain genetic and lifestyle reasons for cancer to the player and emphasize the importance of early detection and screening. Level 2 introduced basic cancer knowledge by demonstrating what was happening inside the patient’s body during stage 4 cancer (Figure 2).

**Figure 3:**
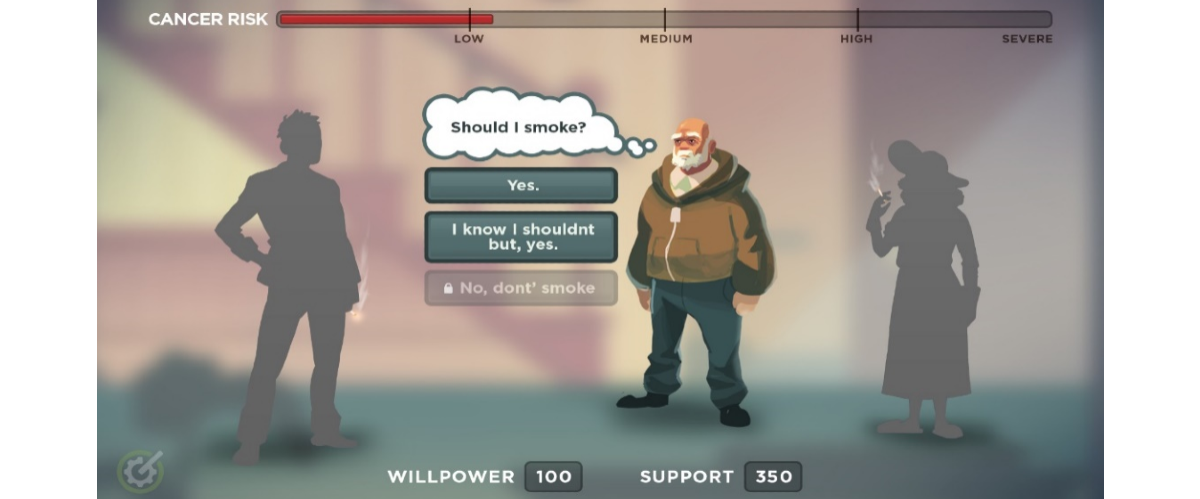
Level 3 of the game playbook.

In Level 3, the player is given the opportunity to learn about cancer risk and prevention through time travel (Figure 3). The goal was to identify what the player could do differently to reduce their risk of cancer.

**Figure 4:**
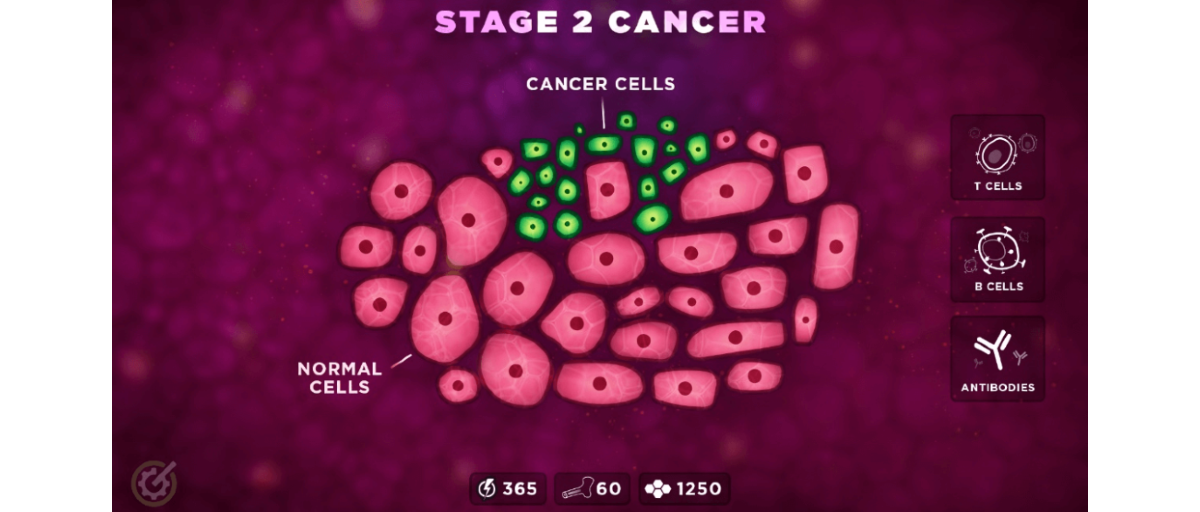
Level 4 of the game playbook.

In the final level, the player is transported back to the present (Figure 4). The player is presented with a view inside the patient’s body again, this time demonstrating stage 2 cancer and an opportunity to play as the immune system, combating cancer. The player learns that, due to early detection, they can survive their cancer diagnosis.

### Sampling and Recruitment

The participants were recruited from a US Midwest middle school in March 2020. Students were eligible if they were 12 to 14 years old, could speak and understand English, and if their parents could read English or Spanish. The study team members worked with school staff to develop a recruitment and data collection plan that met the needs of the school setting. The school staff distributed packets containing a letter of introduction to the study and consent forms to all students in a required 8th grade health class. All consent documents were available in English and Spanish. Parent or guardian consent was required for participants under the age of 18 years. This study was approved by the University’s Institutional Review Board. The participants were each given $10 in cash after participation in the focus groups.

### Data Collection

Each focus group consisted of 5 to 10 participants and was facilitated by 1 to 3 members of the study team. Of the 343 eligible students, 148 (43%) consented to participate in the study. A total of 139 students who consented were present for data collection. Schools provided separate rooms for each focus group to ensure privacy for the participants. One study team member led the focus group discussion, while the others took observation notes and asked additional follow-up questions as needed. The focus groups lasted approximately 35 to 50 minutes each, were audio-recorded, and were professionally transcribed verbatim. The facilitators and moderators completed reflection notes at the end of each focus group.

### Data Analysis

Transcripts were independently verified for accuracy and quality of transcription by 2 members of the study team before beginning data analysis. The transcripts were analyzed content-wise and thematically by 2 study team members using the NVivo 12 (QSR International) qualitative software [19-22]. Codes were developed using an inductive and deductive approach. Each team member independently reviewed and coded transcripts to develop relevant codes, which were combined to create a master codebook. Two team members then completed coding using the master codebook and code definitions and held weekly meetings to review coding and address discrepancies. The intercoder reliability (kappa coefficient) among the 2 coders was reported as 0.97. The prevalent codes were identified by the research team and categorized into major themes and subthemes.

## Results

### Participant Demographic Characteristics

A total of 18 focus groups were held with 139 participants. The reasons for nonparticipation include absence on the day of study activities, participants forgetting to provide their parents with the recruitment packets or to bring back signed forms, lack of signed parental consent, or unknown factors such as time constraint or research burden. All participants were 8th grade students at a Wisconsin middle school. Table 1 summarizes participant characteristics.

The study participant demographics were similar to past years’ student demographics at the school [23]. More than half of the participants were male (54% [n=75]), White (89.9% [n=125]), and 14 years of age (54% [n=75]). Moreover, 39% (n=54) of the participants had 2 other people under the age of 18 living at home with them, while nearly one-third (28% [n=39]) had 3 other people under the age of 18 living at home with them. Three major themes were identified in the focus groups, which were (1) educational video games, (2) game content, and (3) purpose of game (Table 2).

**Table 1:** Participant demographics (N=139).

Characteristics			Values, n (%)
**Gender^a^**			
	Male	75 (54.0)	
	Female	63 (45.3)	
**Age^b^ (years)**			
	12	1 (0.7)	
	13	60 (43.2)	
	14	75 (54.0)	
**Number of youths living at home**			
	1	19 (13.7)	
	2	54 (38.8)	
	3	39 (28.1)	
	4	18 (12.9)	
	5	5 (3.6)	
	6	4 (2.9)	
**Race or ethnicity^c^**			
	American Indian or Native American	2 (1.4)	
	Asian	6 (4.3)	
	Black or African American	5 (3.6)	
	Hispanic or Latino	8 (5.8)	
	Native Hawaiian or Other Pacific Islander	1 (0.7)	
	White	125 (89.9)	

^a^Gender was missing for 1 participant. An option for “Other” was provided but not selected by any participant.

^b^Age was missing for 3 participants.

^c^Race or ethnicity was missing for 1 participant. Race was not a mutually exclusive choice for 8 participants. An option for “Other” was provided but not selected by any participant.

**Table 2:** Themes, subthemes, and verbatim quotes.

Theme and subtheme			Verbatim quotes
**Educational video games**			
	Overall perceptions	*I think it's a good idea because it really gives you an understanding of what you're learning about, and it's fun at the same time. So it really makes kids want to go back to it*. [Male participant, focus group J]	
	Recommended settings	*I also think it could be a good study tool, you know. If it were to be, if we were to have a test on cancer and cancer prevention and all that stuff, I think a game like this could be super helpful in that because then you could still have fun and learn*. [Female participant, focus group N]	
	Recommended platforms	*Computers, definitely, because we have them supplied at our school.* [Male participant, focus group S]	
**Game content**			
	Story lines	*Maybe you get a few choices at the beginning that are kind of in between. They're not really about cancer or anything like that. But depending on how you answer those first few, they could change what you can answer later. Maybe let's say you choose, at one point, to have a certain group of friends. And then later in life, when you go back later, I don't know, it could have where you don't have the choice to not smoke. You have to because of your friend group you chose*. [Male participant, focus group M]	
	Characters	*I think if you could have different options, like different hairstyles or something, and you could just customize it like that, like different things, that would be cool because then you could make it relatable because they could make it more personalized*. [Female participant, focus group K]	
	Educational components	*You could have like the doctor talk about the cancer cells, and like they can describe it instead of just clicking all over*. [Female participant, focus group G]	
**Purpose of game**			
	Cancer education	*I think it's like, the game is a fun way to educate people who don't really know about cancer*. [Female participant, focus group O]	
	Cancer prevention	*[The purpose is] to educate school-aged children on the choices they can make now that can help prevent cancer in the future*. [Female participant, focus group K]	

### Participant Gaming Experience and Preferences

The study participants had varying levels of gaming experience, ranging from “a little” to “a great deal.” Approximately 17% (n=23) had “a little,” 46% (n=63) had “some,” 26% (n=36) had “a lot,” and 15% (n=20) had “a great deal” of experience. The reasons for not playing games included participation in other activities, not having enough time, and use of social media such as Snapchat and TikTok.

Combat was most often reported as the participants’ favorite video game genre, followed by sports, adventure, racing, and strategy. The participants preferred the combat genre due to its competitive, adventurous, and challenging nature. They stated that they played video games such as Call of Duty, Madden, Fortnite, NBA 2K, and Rainbow Six Siege, and mobile games, including Clash of Clans, Clash Royale, and Slope. The participants reported using video game platforms such as Xbox, Wii, Nintendo Switch, and PlayStation. Most participants identified mobile phones as their preferred platform for gaming. The participants also mentioned playing card or tabletop games. The most frequently mentioned tabletop games included Monopoly, Life, and Sorry, and the most reported card games were Uno, Cards Against Humanity, and Poker.

### Theme 1: Educational Video Games

#### Overall Perceptions

The study participants stated that SGs could be used to teach players about cancer through active engagement. Games can offer an entertaining way to visually learn information, thereby making it easier to remember. SGs were preferred over lectures, books, videos, and websites for educational purposes.

School is really stressful, and you get this time to have a little bit of fun while still learning the stuff you need to learn. And I think that if we are able to learn that way, I don't know why the teachers don't let us. It will stick with you.

Female participant, focus group B

#### Recommended Settings

The participants reported that SGs about cancer would be useful in school and for individuals who know someone with cancer. They suggested the use of video games in school, such as in health class, guidance counseling, and test preparation.

As opposed to making us sit through a 30-minute video or read a bunch of websites, I think this [educational video game] would be much more appreciated to learn from at school.

Female participant, focus group D

#### Recommended Platforms

The participants expressed a desire for SGs to be compatible for use on multiple devices or platforms, such as mobile phones and computers.

I feel like in school, computer would be better. But then outside of school, a phone would be a lot more accessible.

Male participant, focus group D

### Theme 2: Game Content

#### Story Lines

The participants preferred an SG that enables players to make choices for their characters. They suggested that in-game decision-making should carry consequences and affect the characters’ outcome. Options could include making healthier dietary choices, resisting peer pressure to smoke, and having regular exercise. The study participants also desired more details provided in the story for context and to create a more immersive experience.

It would be able to help you really get immersed in the game and all the experiences you could feel and all the choices you’d make would have their own consequences, whether good or bad.

Male participant, focus group A

The participants suggested using a story line that included a fight scene between cancer cells and the body’s own immune cells to make the game more interactive. However, some participants indicated that this may only be appealing to a younger audience. The inclusion of some lifestyle choices was recommended.

I think that we should make an actual fighting level for the cancer so kids would be more interacting with the game, so they don't just watch what happens. They can choose, I don't know, to actually fight the cancer cells with the normal cells.

Male participant, focus group F

#### Characters

The ability to customize the main character was discussed in all focus groups. The participants stated that the ability to design a character that looks like them makes a game more engaging. This includes customizing various features such as the character’s age, gender, hair, and clothes.

I think if you had a character customization thing, so at the beginning, you can choose the gender and then all the attributes about him, the age and stuff, so that it could be as much as you or your family member or whoever.

Male participant, focus group H

Secondary characters discussed in the focus group included a doctor, family, and friends. The doctor was the most frequently discussed secondary character. The participants explained that a doctor could serve as an informational resource for the player and could teach players about cancer. Family and friends were similarly considered pertinent to the story and could be used to illustrate the effects a cancer diagnosis may have on loved ones or create peer pressure to make decisions more complex.

I think it’s a good idea to have the character of a doctor explaining it [information about cancer] rather than just a normal person.

Male participant, focus group H

#### Educational Components

The participants expressed their desire to learn about different types of cancers (eg, colorectal, lung, skin, and breast) through video games. They expressed interest in having different types and stages of cancers incorporated into different levels in the game. They also suggested including information about the various types of cells involved, cancer stages, and other facts about cancer.

I think different characters could have different types of cancers in different stages.

Female participant, focus group O

### Theme 3: Purpose of the Game

#### Cancer Education

Cancer education was identified as the main purpose of the game prototype. The participants thought the game could be used as an educational tool to help the player learn about cancer because it allowed the player to visualize different aspects of the disease, including its causes, pathophysiology, and the emotional repercussions on friends and family.

Cancer is not fun, but it [the educational video game] gives you a fun way to actually learn about it. It's not just, oh, all this happens. It gives you visuals, and you actually get to make the choices and see the effects of it.

Female participant, focus group B

#### Cancer Prevention

The participants also identified cancer prevention as a main purpose of the game prototype. They stated that the game could be used to highlight modifiable cancer risk factors so players could minimize these risk factors in their own lives. Additionally, the participants recognized the importance of forming healthy habits at a younger age and affirmed that the game could equip them to do so as well.

It will teach about cancer and cancer prevention because it's showing you things that you can do to prevent cancer, and it's also kind of showing you some more specific things about the cancer cells itself.

Female participant, focus group N

## Discussion

### Principal Findings

An SG for cancer-related education was well received by most participants across the focus groups in this study. The participants desired the ability to customize the main character to look like themselves. Customizing a game character is a popular feature in video games because it increases the player’s engagement, thereby creating a more immersive experience, which may make the game more enjoyable [24-26]. Secondary characters, such as a doctor, family, and friends, were favored to teach the players about cancer. Secondary characters may also contribute to the educational experience by evoking an emotional response when the players observe the effects of their choices. The participants reported that SGs provide an entertaining method for cancer education, thus improving the likelihood of retaining what they learned. Additionally, the participants desired the SG to be used as a supplemental learning material during classes in school.

The participants preferred settings and story lines that mirror real life situations, which could also increase engagement and attachment to the gaming experience. Additionally, they suggested that SGs would be useful in the school setting and preferred SGs over other educational materials, such as lectures, books, and websites. Engaging with the material presented in the game by evaluating choices and decision-making promotes active learning and can be a more effective tool than passively reading text or listening to a lecture. Active learning provides opportunities for discussion and critical thinking that can be meaningful for both students and teachers [27]. In addition to the promotion of active learning, the ability to make choices for characters in the games can encourage students to explore their own perceptions and beliefs about the decisions they might make in the future or under peer pressure [28-30]. Realistic scenarios can bring to life the nuances and complexities that go into the decisions people make in their daily lives and encourage behavior change [28-30].

Overall, 87% of the participants stated they had at least “some” to “a great deal” of gaming experience. This is similar to national trends, which found that approximately 90% of adolescents play video games and 80% of teenagers have access to gaming devices [6]. Although the participants described their favorite video game genre as combat, they preferred an SG for cancer education to follow a more realistic story line that offers decision-making opportunities. Additionally, many participants stated they want an SG to be accessible across multiple platforms, such as computers, mobile phones, and video game consoles. Games accessible by computer would be beneficial for use in schools, while mobile games would allow older children and adolescents to engage in gameplay at home as well.

Our study provides middle school students’ support for the use of an SG as an educational tool for cancer prevention education. An SG can simulate real-life situations in which making healthier decisions may be difficult due to social pressures or other barriers. An SG can provide players with a visual tool for learning about cancer and cancer prevention through the illustration of modifiable risk factors set in realistic scenarios the players may encounter in their own lives. Therefore, the SG could provide youth with the tools needed to make healthy behavior choices in real life. Future research should examine the effect an SG has on adolescents’ cancer awareness, knowledge, and mindfulness of lifestyle choices.

### Limitations

This study was conducted at a single Midwestern middle school. While the demographics of the study participants mirror the demographics of this school, the responses gathered from this sample may not be representative of the general United States older children and adolescent population. Future studies should explore preferences of a larger and more representative sample of middle school students for an SG on cancer education and incorporate their feedback into the design of a game prototype.

### Conclusions

This study suggests that children and adolescents consider SGs an entertaining tool for education about cancer prevention and associated healthy lifestyle habits, particularly in the school setting. The study participants stated that SGs can offer a “real-life” virtual immersion experience with rewards and consequences of health-based lifestyle choices. Many participants reported customizing the main characters would enable them to partake in the gaming experience and emotionally connect with the outcomes. Additionally, the participants favored an SG that incorporated realistic settings and story lines, customizable characters, and information about various cancer types. Older children and adolescent preferences should be considered in the process of designing an SG for cancer education to create a game that is engaging and acceptable for youth.

## Appendix

Multimedia Appendix 1 Focus group guide.

## Abbreviations

SG: serious game

## References

[1] Siegel RL, Miller KD, Fuchs HE, Jemal A (2021). Cancer Statistics, 2021. CA A Cancer J. Clin.

[2] Mariotto Angela B, Enewold Lindsey, Zhao Jingxuan, Zeruto Christopher A, Yabroff K Robin (2020). Medical Care Costs Associated with Cancer Survivorship in the United States. Cancer Epidemiol Biomarkers Prev.

[3] Howlader N, Noone A, Krapcho M, Miller D, Brest A, Yu M, Ruhl J, Tatalovich Z, Mariotto A, Lewis D, Chen H, Feuer E, Cronin K SEER Cancer Statistics Review (CSR), 1975-2018. National Cancer Institute.

[4] Robalino JD, Macy M (2018). Peer effects on adolescent smoking: Are popular teens more influential?. PLoS ONE.

[5] Islami F, Goding Sauer A, Miller KD, Siegel RL, Fedewa SA, Jacobs EJ, McCullough ML, Patel AV, Ma J, Soerjomataram I, Flanders WD, Brawley OW, Gapstur SM, Jemal A (2017). Proportion and number of cancer cases and deaths attributable to potentially modifiable risk factors in the United States. CA: A Cancer Journal for Clinicians.

[6] (2108). Teens, Social Media and Technology 2018. Pew Research Center.

[7] Alonso-Fernandez C, Calvo A, Freire M, Martinez-Ortiz I, Fernandez-Manjon B (2017). Systematizing game learning analytics for serious games.

[8] Ohannessian R, Yaghobian S, Verger P, Vanhems P (2016). A systematic review of serious video games used for vaccination. Vaccine.

[9] DeShazo J, Harris L, Pratt W (2010). Effective intervention or child's play? A review of video games for diabetes education. Diabetes Technol Ther.

[10] Abraham O, LeMay S, Bittner S, Thakur T, Stafford H, Brown R (2020). Investigating Serious Games That Incorporate Medication Use for Patients: Systematic Literature Review. JMIR Serious Games.

[11] Kato PM, Cole SW, Bradlyn AS, Pollock BH (2008). A video game improves behavioral outcomes in adolescents and young adults with cancer: a randomized trial. Pediatrics.

[12] Khalil GE, Beale IL, Chen M, Prokhorov AV (2016). A Video Game Promoting Cancer Risk Perception and Information Seeking Behavior Among Young-Adult College Students: A Randomized Controlled Trial. JMIR Serious Games.

[13] Doll R, Hill AB (1950). Smoking and Carcinoma of the Lung. BMJ.

[14] Van Parijs LG (1986). Public education in cancer prevention. Bull World Health Organ.

[15] Barros A, Santos H, Moreira L, Ribeiro N, Silva L, Santos-Silva F (2015). The Cancer, Educate to Prevent Model—the Potential of School Environment for Primary Prevention of Cancer. J Canc Educ.

[16] Then KL, Rankin JA, Ali E (2014). Focus group research: what is it and how can it be used?. Can J Cardiovasc Nurs.

[17] Fredrick CM, Linskens RJ, Schilling MA, Eggen AT, Strickland RA, Jacobs EA (2020). J Canc Educ.

[18] Cancer Health Disparities Initiative.

[19] Krippendorff K (2018). Content Analysis An Introduction to Its Methodology (4th Edition).

[20] Rabiee F (2007). Focus-group interview and data analysis. Proc. Nutr. Soc.

[21] Elo S, Kyngäs H (2008). The qualitative content analysis process. J Adv Nurs.

[22] Houghton C, Murphy K, Meehan B, Thomas J, Brooker D, Casey D (2017). From screening to synthesis: using nvivo to enhance transparency in qualitative evidence synthesis. J Clin Nurs.

[23] Waunakee Middle. U.S. News & World Report.

[24] Fischer P, Kastenmüller A, Greitemeyer T (2010). Media violence and the self: The impact of personalized gaming characters in aggressive video games on aggressive behavior. Journal of Experimental Social Psychology.

[25] Teng C (2010). Customization, immersion satisfaction, and online gamer loyalty. Computers in Human Behavior.

[26] Brown E, Cairns P (2004). A grounded investigation of game immersion. CHI '04 Extended Abstracts on Human Factors in Computing Systems.

[27] Torralba KD, Doo L (2020). Active Learning Strategies to Improve Progression from Knowledge to Action. Rheumatic Disease Clinics of North America.

[28] Kaczmarczyk J, Davidson R, Bryden D, Haselden S, Vivekananda-Schmidt P (2015). Learning decision making through serious games. Clin Teach.

[29] Lieberman D (2015). Using Digital Games to Promote Health Behavior Change. The Handbook of the Psychology of Communication Technology.

[30] Hieftje K, Edelman EJ, Camenga DR, Fiellin LE (2013). Electronic media-based health interventions promoting behavior change in youth: a systematic review. JAMA Pediatr.

