# Supplementary material for: Exploring Middle School Students’ Perspectives on Using Serious Games for Cancer Prevention Education: Focus Group Study
Source: JMIR Serious Games. 2022 Jan 24;10(1):e31172. doi: 10.2196/31172 (PMC8822422; doi:10.2196/31172)
Supplement: Multimedia Appendix 1 [file games_v10i1e31172_app1.docx]

**Multimedia Appendix 1.** Focus group guide.

*BEFORE STARTING, HAND OUT NAME TAGS AND DIRECT STUDENTS TO COME UP WITH FAKE NAMES TO REFER TO EACH OTHER*

**Thank you all for being here. My name is __________, and this is my co-moderator, __________, and we will be leading this focus group today.**

**As mentioned in the consent document, we will be recording this focus group conversation. Your personal information will remain confidential, and none of these records will be linked back to you. Please avoid saying your name or the names of others in this group. We also ask that you avoid saying the names of any cities, teachers, schools, classmates, or family members.**

**The purpose of this focus group is to learn your thoughts about the draft of a video game we are designing. We are very interested in hearing your thoughts and ideas so that we can create better educational materials for people your age.**

**Feel free to ask questions and feel free to not answer any questions if you feel uncomfortable answering them***.* **Lastly, since this conversation will be recorded, we ask that you try to speak loudly and avoid speaking over each other as much as possible. What questions can we answer before we start the focus group?**

*TURN ON RECORDER*

**Before we begin, let’s have you all say your fake names for the recorder. We’ll start to my left and work counter-clockwise around the room.**

*MODERATORS WILL DISPLAY OR HAND OUT GAME PLAYBOOK AND HAVE STUDENTS REVIEW GAME ELEMENTS. (DISPLAY PIECES ONE-BY-ONE AS QUESTIONS ARE ASKED—START WITH CHARACTERS, THEN MOVE ON TO SETTING/STORYLINE/ETC)*

CHARACTERS

**What are your thoughts about the characters?**

**What do you like about the characters?** *(PROMPT: ARE THEY RELATABLE? DO YOU LIKE THE DESIGN? THEIR BACKGROUND? ETC)*

**Is there any character that you like more than others? IF YES: Which character? Why do you like this character?**

**What do you dislike about the characters?**

**Is there any character that you dislike more than others? IF YES: Which character? Why do you dislike this character?**

**Are there any characters not in the game that would be interesting to add? IF YES: Please describe it/them. Why would you add it/them?**

STORYLINE/SCENARIOS

**How do you feel about the overall storyline for the game?**

**Do the scenarios seem realistic or unrealistic? What makes them realistic/unrealistic?**

**Are there any scenarios not in the game that would be interesting to add? IF YES: Please describe them. Why would you add them?**

SUCCESS OF GAME

**What is the purpose of this game in your view? Why do you see this as the purpose?**

**What would help the game meet its main goal?**

**Do you think this game could help you learn about cancer and cancer prevention? Why or why not?**

**Would you recommend a game like this to your friends? Why/why not?**

**What unanswered questions do you have about the game?**

EXPERIENCE WITH VIDEO GAMES

**How much experience do you have playing video games: no experience at all, a little experience, some experience, a lot of experience, or a great deal of experience?**

IF RESPONSE IS: NO OR A LITTLE EXPERIENCE WITH VIDEOGAMES:

**Is there a particular reason you don’t play video games? IF YES: What is it?**

**Is there anything that could be changed about video games that would make you want to play them? IF YES: What is it?**

**Do you play any mobile games?**

**Do you play any table top games?**

IF RESPONSE IS: SOME OR MORE EXPERIENCE WITH VIDEOGAMES:

**There are different types of video games – sports, adventure, racing, combat, etc. Do you prefer one type of videogame more than the others? IF YES: Which type?**

**What video games are you playing these days?**

**Is using videogames for educational goals a good idea or a bad idea? Why?**

**Have you ever played any kind of videogame that had an educational goal? IF YES: What was it? Did that videogame have any features that could improve the video game you played today?**

WRAP-UP

**Before we finish this focus group, does anyone have anything they’d like to add?**

**Thank you so much for participating in this focus group today. The information you shared will be very helpful in developing better educational materials for people your age.**

*TURN OFF RECORDER*
